# Complete Genome Sequence of Lactococcus lactis AH1, Isolated from Viili, a Finnish Dairy Product

**DOI:** 10.1128/mra.00318-22

**Published:** 2022-06-15

**Authors:** Pavelas Sazinas, Jan Martinussen

**Affiliations:** a Metabolic Signaling and Regulation Group, DTU Bioengineering, Technical University of Denmark, Lyngby, Denmark; University of Maryland School of Medicine

## Abstract

Here, we report the complete genome sequence of Lactococcus lactis strain AH1, isolated from viili, a Finnish dairy product. This strain is known for the extreme viscosity it imparts to fermented milk due to its production of exopolysaccharides. The complete sequence was obtained by combining Illumina and Nanopore data, revealing a chromosome with a length of 2,421,519 bp and eight plasmids ranging from 5,773 to 55,958 bp.

## ANNOUNCEMENT

Some dairy products are characterized by an extremely high viscosity due to the high production of microbial exopolysaccharides. Examples include the fermented milk products from northern Scandinavia known as viili or langfil. The bacterial strains used for these products are typically *Lactococci* (1). Here, we have isolated a strain from viili and report the assembled sequence of its entire genome, including the chromosome and eight plasmids.

To isolate an exopolysaccharide-producing Lactococcus lactis strain, a viili sample was obtained. A freeze-dried viili starter was bought from Yemoos, diluted in 0.9% sodium chloride, and plated onto M17 medium (Oxoid, Basingstoke, UK) supplemented with 1% lactose. After 24 h at 30°C, single colonies were purified, and one was stocked as L. lactis AH1. For extraction of genomic DNA from L. lactis AH1 using the Easy-DNA kit (catalog number K1800-01; Invitrogen), a single colony was inoculated in M17 broth with 1% glucose and grown overnight at 30°C. Genomic libraries were prepared using the Nextera XT DNA sample preparation kit (Illumina, San Diego, CA; catalog number FC-131-1024) and then sequenced using v3, 2 × 300-bp chemistry on the Illumina MiSeq platform. For Nanopore sequencing, the genomic DNA was purified using the PureLink genomic DNA kit (Thermo Fisher Scientific); DNA libraries were prepared using the rapid sequencing kit (Oxford Nanopore, Oxford, UK) and sequenced on a MinION instrument using a FLO-MIN106 flow cell. In total, 2,999,192 paired-end Illumina reads were generated and subsequently trimmed based on quality (Q20) using Trimmomatic v0.38. MinKNOW v19.12.2 (Oxford Nanopore) was used to base call 199,948 Nanopore reads with a value greater than Q10 (average read length, 2,799 bp; read length *N*_50_, 5,172 bp). Read quality checks were performed using FastQC v0.11.7 and NanoStat v1.1.2. Unicycler v0.4.6 was used to carry out a hybrid genome assembly from both the Illumina and Nanopore reads. A complete circular chromosome (2,421,519 bp) was assembled and rotated using Unicycler, with the starting gene being *dnaA*. Moreover, eight complete circular plasmids ranging from 5,773 to 55,958 bp were found (Table 1).

**TABLE 1:** Assembly and annotation of the total DNA sequence of Lactococcus lactis AH1

Name	DNA type	GenBank accession no.	Length (bp)	GC content (%)	No. of annotated coding sequences
AH1	Chromosome	CP093413	2,421,519	35.3	2,376
pAH1-1	Plasmid	CP093414	55,958	36.5	63
pAH1-2	Plasmid	CP093415	49,492	34.8	54
pAH1-3	Plasmid	CP093416	49,443	33.0	55
pAH1-4	Plasmid	CP093417	30,305	35.1	30
pAH1-5	Plasmid	CP093418	11,236	32.7	10
pAH1-6	Plasmid	CP093419	8,277	34.8	12
pAH1-7	Plasmid	CP093420	8,084	31.1	8
pAH1-8	Plasmid	CP093421	5,773	34.0	5

The chromosome and plasmid sequences were submitted to NCBI and subjected to the associated annotation pipeline, PGAP. A BLASTN search, using the 16S rRNA sequence as the query, confirmed that the strain indeed was a Lactococcus lactis strain, as 100% identity to several Lactococcus lactis strains was found. Moreover, the annotation suggested that the genes relevant for exopolysaccharide biosynthesis were plasmid-encoded on pAH1-3 and associated with several insertion sequences. Default software parameters were used throughout unless otherwise stated.

### Data availability.

The genome sequence of Lactococcus lactis strain AH1 has been deposited at GenBank under the accession numbers posted in Table 1. The associated BioProject accession number is PRJNA814528, whereas the raw sequence data are available under the SRA accession number SRR18888551.

## References

[1] Macura D, Townsley PM. 1984. Scandinavian ropy milk—identification and characterization of endogenous ropy lactic streptococci and their extracellular excretion. J Dairy Sci 67:735–744. doi:10.3168/jds.S0022-0302(84)81363-6.

